# Brown Sequard

**Published:** 1869-03

**Authors:** 


Brown-Séquard.—Dr. Brown-Séquard has accepted a chair in the Paris Faculty of Medicine.

